# Interleukin 1 Polymorphisms Contribute to Intervertebral Disc Degeneration Risk: A Meta-Analysis

**DOI:** 10.1371/journal.pone.0156412

**Published:** 2016-06-02

**Authors:** Zheng Wang, Zhigang Qu, Changfeng Fu, Feng Xu, Yong Chen, Zhenyu Wang, Yi Liu

**Affiliations:** 1 Department of Spinal Surgery, The First Hospital of Jilin University, No.71, Xinmin Avenue, Chaoyang District, Changchun, Jilin Province, 130021, China; 2 Department of Neurosurgery, The First Hospital of Jilin University, No.71, Xinmin Avenue, Chaoyang District, Changchun, Jilin Province, 130021, China; Indiana University Bloomington, UNITED STATES

## Abstract

**Objective:**

We performed a meta-analysis to assess association between interleukin 1 (IL-1) polymorphisms and the risk of Intervertebral Disc Degeneration (IDD).

**Background:**

A series of studies have investigated the association between common single nucleotide polymorphisms in IL-1 and IDD risk; however, the overall results are inconclusive.

**Methods:**

Two independent investigators conducted a systematic search for relevant available studies. Allele frequencies were extracted from each study. The association between the IL-1α (+889C/T) or IL-1β (+3954C/T) polymorphism and IDD risk was measured by odds ratios (OR) with 95% confidence intervals (95% CI).

**Results:**

Five and six studies, respectively, were ultimately included in the meta-analysis for the IL-1α (+889C/T) and IL-1β (+3954C/T) polymorphism. The combined results showed that the IL-1α (+889C/T) polymorphism was significantly associated with increased susceptibility to IDD, particularly in Caucasians (TT versus CC: OR = 2.95, 95% CI: 1.45, 6.04; *P*_heterogeneity_ = 0.82; TT versus CC/CT: OR = 2.29, 95% CI: 1.18, 4.47; *P*_heterogeneity_ = 0.20). In contrast, the IL-1β (+3954C/T) polymorphism showed a trend towards increased risk in Caucasians but no association in Asians.

**Conclusion:**

This meta-analysis suggested that the IL-1α (+889C/T) polymorphism is significantly associated with risk of IDD, especially in Caucasian populations.

## Introduction

Intervertebral disc degeneration (IDD) is a multifactorial disease of the musculoskeletal system [1]. As degeneration increases, so does the risk of lower back pain, disc herniation, and even radiating pain from the sciatic nerve [2]. Because it is a major cause of disability at work, increasing attention has been paid to IDD [3]. The pathogenesis and cause of IDD are not fully elucidated, but epidemiologic studies have suggested that IDD is associated with adverse mechanical, environmental, and genetic factors [4]. The interplay between diverse cytokines, growth factors, and proinflammatory cytokines and their inhibitors, forms an intricate balance between anabolic and catabolic processes [5, 6]. Increased expression of proinflammatory cytokines such as matrix metalloproteases has been observed in degenerating discs; thus, inflammatory factors might play an important role in the pathogenesis of IDD [7]. Further understanding of the pathophysiologic mechanisms of IDD may help identify biological treatments capable of inhibiting inflammatory reactions in intervertebral discs.

Interleukin-1 (IL-1) is a proinflammatory cytokine involved in inflammatory processes and the induction of apoptosis in response to cell injury [8]. The *IL-1* gene family contains three members: IL-1 alpha, IL-1 beta (IL1A, IL1B), and IL-1 receptor antagonist (IL-1RN). Intervertebral discs are known to respond to IL1A and IL1B in the multiple pathological processes of disc degeneration, such as inhibiting synthesis of the extracellular matrix and increasing synthesis of matrix metalloproteinases [9, 10]. IL-1 also can induce histiocytes and produce prostaglandin E2, which causes pain directly and simultaneously increases sensitivity to other pain producers [11]. Maeda and Kokubun showed that IL-1 could contribute to IDD by both decreasing proteoglycan synthesis and increasing cell sensitivity [12]. Rannou et al. showed that, in annulus fibrosus cells, the production of prostaglandin E2 was increased and the secretion of type II phospholipase A2 activity was increased in a dose-dependent manner after IL-1 stimulation [13].

Several studies have investigated the association between IL-1 gene polymorphisms and the risk of IDD, focusing on two particular variants: IL-1α (+889C/T) (rs1800587) and IL-1β (+3954C/T) (rs1143634). These studies have obtained conflicting results. IL-1α (+889C/T) has been significantly associated with disc bulges and Modic changes in an occupational male cohort [14]. Furthermore, IL-1 is the dominant cytokine in the destruction of cartilage and inhibition of proteoglycan synthesis in the intervertebral discs in an animal model. Unlike IL1β, IL1α is already bioactive at the precursor stage [12, 15]. Solovieva et al. found that the IL-1β (+3954C/T) polymorphism affected the risk of disc degeneration and that IL-1 gene polymorphisms could reduce the effect of physical workload [16].

Considering the conflicting results, we felt it worthwhile to summarize the current data on the associations between IL-1 polymorphisms and the risk of IDD. Thus, we performed a meta-analysis from all eligible studies [14, 16–21], to evaluate the association of IL-1 gene polymorphisms with risk of IDD.

## Materials and Methods

### Identification of eligible studies

Two independent investigators conducted a systematic search for relevant available studies published in English or Chinese from four databases (PubMed, Embase, the China National Knowledge Infrastructure database, and the China Biology Medical Literature database). The final literature search was conducted on October 1, 2015. The following terms were used: (“IL-1”or “interleukin-1” “interleukin 1” or “interleukin I” or “T Helper Factor”) and (“polymorphism” or “SNPs” or “Single Nucleotide Polymorphisms” or “Nucleotide Polymorphism, Single”) and (“disc degeneration” or “disc” or “disc herniation” or “intervertebral disc degeneration” or “low back pain”) in combination with genetic variations (“gene polymorphism” or “genetic variation”). For all identified studies, the reference lists of the primary articles and recent reviews were also manually searched.

### Inclusion and exclusion criteria

The following inclusion criteria were used: (1) evaluation of the association between IL-1α (+889C/T) or IL-1β (+3954C/T) polymorphism and the risk of IDD; (2) case-control study; (3) human subjects; and (4) sufficient data provided so that the odds ratios (ORs) and 95% confidence intervals (CIs) could be calculated. Accordingly, the exclusion criteria were defined as: (1) comments, reviews, or animal studies; (2) data overlapping with previous publications; (3) family-based design studies; (4) study with useless data, or genotype frequencies not detailed. Eligible studies was reviewed independently by two investigators, according to the inclusion criteria. For any disagreements, a consensus was achieved after discussion.

### Data extraction

Two investigators independently extracted the data from the eligible studies. The following information was collected: (1) name of the first author; (2) year of publication; (3) country where the study was conducted; (4) ethnicity of the study population; (5) gender and age of enrolled subjects; (6) numbers of cases and controls; (7) genotyping method; and (8) genotype frequency in cases and controls. The two authors reached a consensus on all the data.

### Methodological quality assessment

According to the methodological quality assessment scale, which was extracted and modified from previous studies, two authors independently examined the quality of the included studies [22, 23]. On this scale, five items were carefully checked: the representativeness of cases, source of controls, sample size, quality control of genotyping methods, and Hardy-Weinberg equilibrium. The quality score ranged from 0 to 10, with a high score representing a good quality study. Disagreements were resolved by discussion.

### Statistical analysis

Our meta-analysis was conducted according to the PRISMA checklists and followed their guidelines [24]. The association strength between IL-1α (+889C/T) or IL-1β (+3954C/T) polymorphism and IDD risk was measured by OR with 95% CI. The estimates of pooled ORs were determined by the weighted average OR from each study. The significance level was determined by Z-test with a *P*-value less than 0.05 indicating a significant risk. The pooled ORs were calculated respectively for homozygote comparison (TT versus CC), heterozygote comparison (CT versus CC), dominant (CT/TT versus CC), and recessive (TT versus CC/CT) models, assuming dominant or recessive effects of the variant T allele. Furthermore, subgroup analyses were conducted after stratifying by ethnicity.

Statistical heterogeneity was evaluated by the Q statistic and I^2^ statistic, and heterogeneity was considered significant when *P* < 0.10 and I^2^ > 50% [25]. According to the heterogeneity, a fixed-effects or random-effects model was used to pool the effect sizes. The random-effects model was used when there was significant heterogeneity; in other cases, the fixed-effects model was applied [26]. Sensitivity analyses were performed to evaluate the effect of an individual study on the combined ORs by omitting each study in turn. Publication bias was checked with Begg’s funnel plot and Egger’ regression test. An asymmetric plot and *P* < 0.05 on Egger’s test were considered significant [27, 28]. R software was used to run Goodness-of-fit test to analyze data. Goodness-of-fit test was used to check how the models fit the data in meta-analysis [29], where Shapiro-Wilk test was applied in R. p_sw is the p-value that obtained by Shapiro-Wilk test with B = 100000 bootstraps. All analyses were performed using Review Manager 4.2 (Cochrane Collaboration, Oxford, UK) and STATA 12 (Stata, CollegeStation, TX) and R version 3.2.3(UOA, Nz). All *P*-values were two-sided.

## Results

### Characteristics of studies

**Fig 1:**
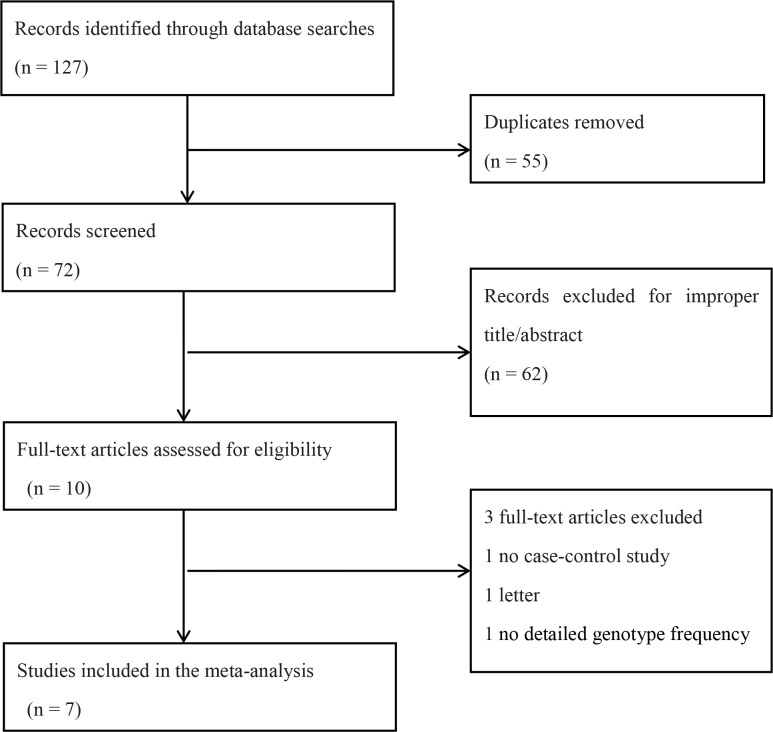
Flow chart of study selection.

The literature selection process is shown in Fig 1. A total of 127 studies concerning IL-1 polymorphisms and IDD risk were acquired from the four databases. Of these, 55 duplicate articles were excluded. A further 62 studies were excluded after reading the titles and abstracts. We evaluated the full text of the ten remaining studies, and excluded three more. Among the excluded articles, one was not a case-control study [30] and two contained no detailed genotype frequencies [31, 32]. Ultimately, seven studies that met the inclusion criteria were included in the meta-analysis [14, 16–21]. In four of these [14, 16, 17, 19], the genotype frequencies of IL-1α (+889C/T) and IL-1β (+3954C/T) were presented separately; therefore, we treated each as separate studies. In total there were five studies for the IL-1α (+889C/T) polymorphism, containing 330 cases and 419 controls, and six studies for the IL-1β (+3954C/T) polymorphism, involving 610 cases and 1007 controls. For the IL-1α (+889C/T) polymorphism, four studies examined individuals of Caucasian descent [14, 16, 17, 19], while the remaining study recruited from a Mexican Mestizo population [20]. As for the IL-1β (+3954C/T) polymorphism, four studies were carried out in Caucasians [14, 16, 17, 19] and the other two in Asians [18, 21]. Blood samples were used for genotyping in all studies. Magnetic resonance imaging was used to diagnose IDD in all the cases. In all seven studies, the controls were in Hardy-Weinberg equilibrium. The detailed characteristics of each study are shown in **Table 1**.

**Table 1:** Characteristics of individual studies for associations between IL-1 polymorphisms and IDD risks.

Authors	Year	Country	Ethnicity	Gender	Age^a^	Genotypes distribution						Quality
						Case			control			
						CC	CT	TT	CC	CT	TT	
**IL-1α(+889C/T)**												
**Aparicio**	2011	Spain	Caucasian	both	44	22	25	3	63	61	5	7
**Karppinen**	2009	Finland	Caucasian	men	44	12	26	7	30	28	5	7
**Serrano**	2014	Mexico	Mexican population	both	39.22	51	45	4	55	35	10	7
**Solovieva(a)**	2004	Finland	Caucasian	men	40–45	11	21	6	34	51	8	6
**Solovieva(b)**	2004	Finland	Caucasian	men	44	30	54	13	15	18	1	6
**IL-1β (+3954C/T)**												
**Aparicio**	2011	Spain	Caucasian	both	43.9	30	16	4	76	50	3	7
**Jihong**	2013	China	Asian	men	21.94	283	22	0	525	61	1	8
**Karppinen**	2009	Finland	Caucasian	men	44	20	20	5	32	24	6	7
**Solovieva(a)**	2004	Finland	Caucasian	men	40–45	19	12	4	42	42	9	6
**Solovieva(b)**	2004	Finland	Caucasian	men	44	41	41	12	21	13	1	6
**Wei**	2007	China	Asian	both	42.7	77	4	0	89	12	0	6

^a^ the mean age(year)

### Association between IL-1α (+889C/T) polymorphism and risk of IDD

**Fig 2:**
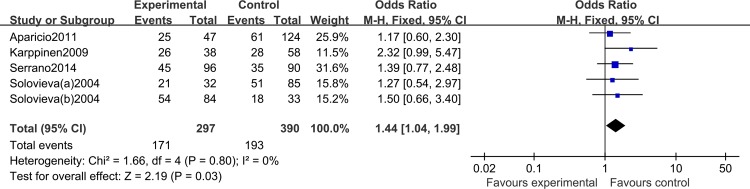
Forest plot of allelic comparison of the IL-1α (+889C/T) polymorphism for overall comparison (CT versus CC).

**Fig 3:**
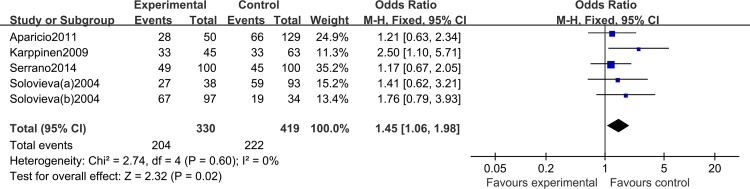
Forest plot of allelic comparison of IL-1α (+889C/T) polymorphism for overall comparison (CT+TT versus CC).

The association between the IL-1α (+889C/T) polymorphism and risk of IDD is shown in **Table 2**. We identified a significantly increased risk of IDD using a heterozygote model (CT versus CC: OR = 1.44, 95% CI: 1.04, 1.99; *P*_heterogeneity_ = 0.22; Fig 2), a dominant model (CT/TT versus CC: OR = 1.45, 95% CI: 1.06,1.98; *P*_heterogeneity_ = 0.40, Fig 3), and when all eligible studies were pooled. In contrast, there was no significant association using a homozygote model (TT versus CC: OR = 1.70, 95% CI: 0.95, 3.04; *P*_heterogeneity_ = 0.76) or recessive model (TT versus CC/CT: OR = 1.41, 95% CI: 0.81, 2.46; *P*_heterogeneity_ = 0.5); however, a trend towards increased risk was noted. After stratification by ethnicity, there was a significant association between the IL-1α (+889C/T) polymorphism and risk of IDD in Caucasians (TT versus CC: OR = 2.95, 95% CI: 1.45, 6.04; *P*_heterogeneity_ = 0.82; TT versus CC/CT: OR = 2.29, 95% CI: 1.18, 4.47; *P*_heterogeneity_ = 0.20). No significant heterogeneity was identified by Q-test and I^2^ statistic under any of the genetic models, so we used a fixed-effects model throughout.

**Table 2:** Meta-analysis Results.

		T vs C		CT vs CC		TT vs CC		CT/TT vs CC		TT vs CC/CT	
	N	OR	P_H_	OR	P_H_	OR	P_H_	OR	P_H_	OR	P_H_
**IL-1α(+889C/T)**											
**Overall**	5	1.30 [1.03, 1.64]	0.85	1.44 [1.04, 1.99]	0.22	1.70 [0.95, 3.04]	0.76	1.45 [1.06, 1.98]	0.40	1.41 [0.81, 2.46]	0.5
**Ethnicity**											
**Caucasian**	4	1.47 [1.12, 1.93]	0.73	1.46 [0.99, 2.16]	0.37	2.95 [1.45, 6.04]	0.82	1.60 [1.09, 2.34]	0.78	2.29 [1.18, 4.47]	0.2
**IL-1β(+3954C/T)**											
**Overall**	6	0.98 [0.68, 1.41]	0.98	0.83 [0.58, 1.19]	0.81	1.29 [0.60, 2.77]	0.76	0.90 [0.61, 1.34]	0.97	1.71 [0.85, 3.46]	0.51
**Ethnicity**											
**Asian**	2	0.61 [0.39, 0.96]	0.32	0.61 [0.38, 0.98]	0.4	0.62 [0.06, 5.95]	NA	0.60 [0.38, 0.96]	0.41	0.64 [0.03, 15.76]	NA
**Caucasian**	4	1.21 [0.88, 1.66]	0.76	1.01 [0.67, 1.53]	0.62	1.41 [0.62, 3.20]	0.63	1.13 [0.74, 1.71]	0.96	1.80 [0.88, 3.70]	0.16

N: number of studies included; OR: odds ratio; Ph: p value for heterogeneity

### Association between IL-1β (+3954C/T) polymorphism and risk of IDD

**Fig 4:**
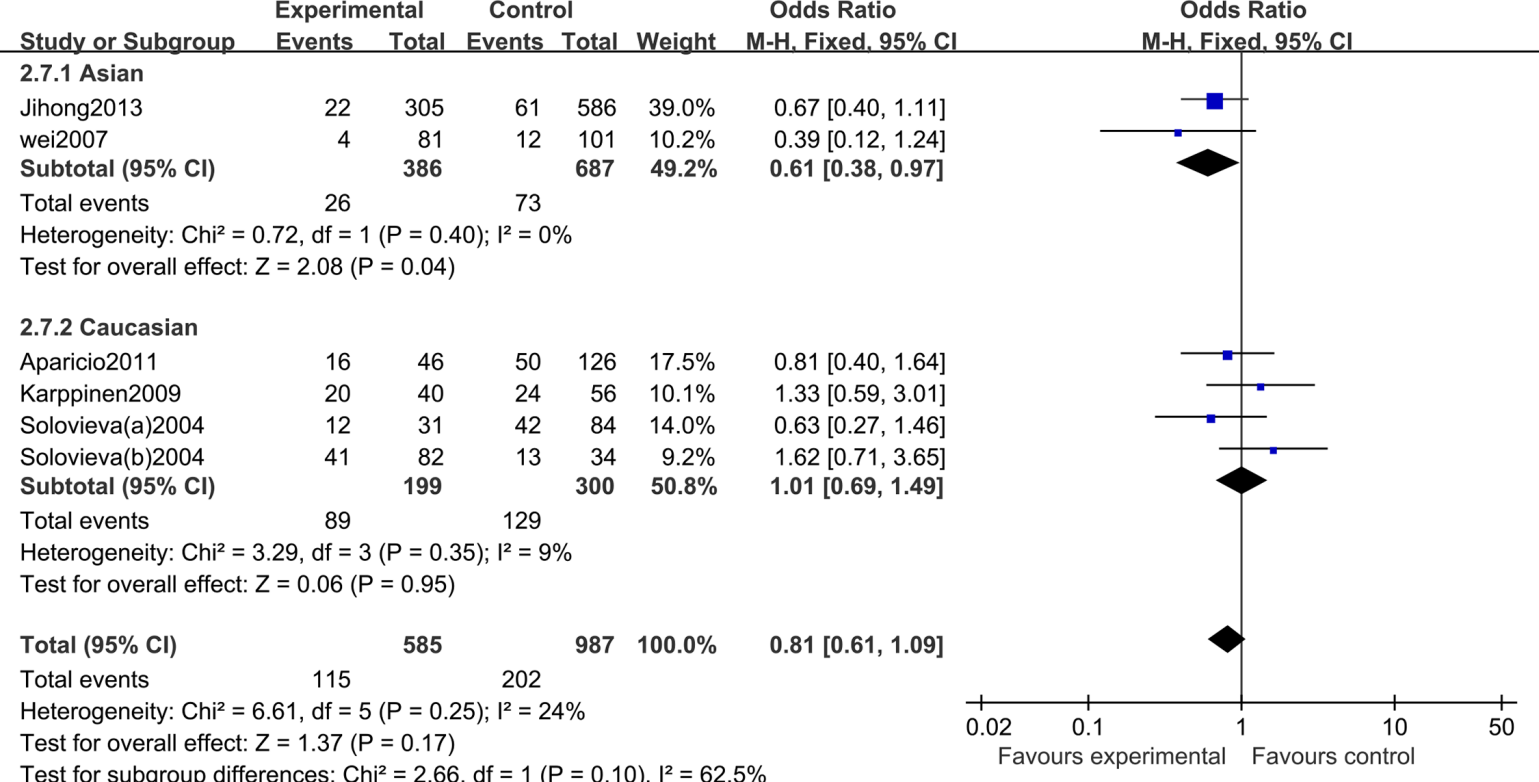
Forest plot of allelic comparison of IL-1β (+3954C/T) polymorphism for overall comparison (CT versus CC).

Meta-analysis of the IL-1β (+3954C/T) polymorphism showed no significant association with risk of IDD in any genetic model, nor even a clear trend towards significance (TT versus CC: OR = 1.29, 95% CI: 0.60, 2.77; *P*_heterogeneity_ = 0.76; CT versus CC: OR = 0.83, 95% CI: 0.58, 1.19; *P*_heterogeneity_ = 0.81; CT/TT versus CC: OR = 0.90, 95% CI: 0.61, 1.34; *P*_heterogeneity_ = 0.97; TT versus CC/CT: OR = 1.71, 95% CI: 0.85, 3.46; *P*_heterogeneity_ = 0.51). Significant heterogeneity was identified by Q-test and I^2^ statistic for the IL-1β (+3954C/T) variant T allele polymorphism. Subgroup analysis stratified by ethnicity revealed a significantly reduced risk of IDD in Asians, using a heterozygote model (CT versus CC: OR = 0.61, 95% CI: 0.38, 0.98; *P*_heterogeneity_ = 0.4; Fig 4) and a dominant model (CT/TT versus CC: OR = 0.60, 95% CI: 0.38, 0.96; *P*_heterogeneity_ = 0.41). As this result was obtained from only two studies, this result should be treated with caution. In Caucasians, we found no significant association under any genetic model (TT versus CC: OR = 1.41, 95% CI: 0.62, 3.20; *P*_heterogeneity_ = 0.63; CT versus CC: OR = 1.01, 95% CI: 0.67, 1.53; *P*_heterogeneity_ = 0.62; CT/TT versus CC: OR = 1.13, 95% CI: 0.74, 1.71; *P*_heterogeneity_ = 0.96; TT versus CC/CT: OR = 1.80, 95% CI: 0.88, 3.70; *P*_heterogeneity_ = 0.16). There was, however, a trend towards increased susceptibility (**Table 2**).

### Sensitivity analysis

We performed sensitivity analysis to examine the influence of each individual study on the pooled ORs by deleting each study one at a time from the pooled analysis. For the IL-1β (+3954C/T) polymorphism, we found that the pooled OR was not changed significantly by the removal of any individual study. However, for the IL-1α (+889C/T) polymorphism, there was a substantial change when excluding a study that comprised subjects of a different ethnicity. The pooled estimates of the remaining four studies showed a significant association using the homozygote and recessive models. This was the same finding as for the previous subgroup analysis according to ethnicity.

### Heterogeneity and publication bias

**Fig 5:**
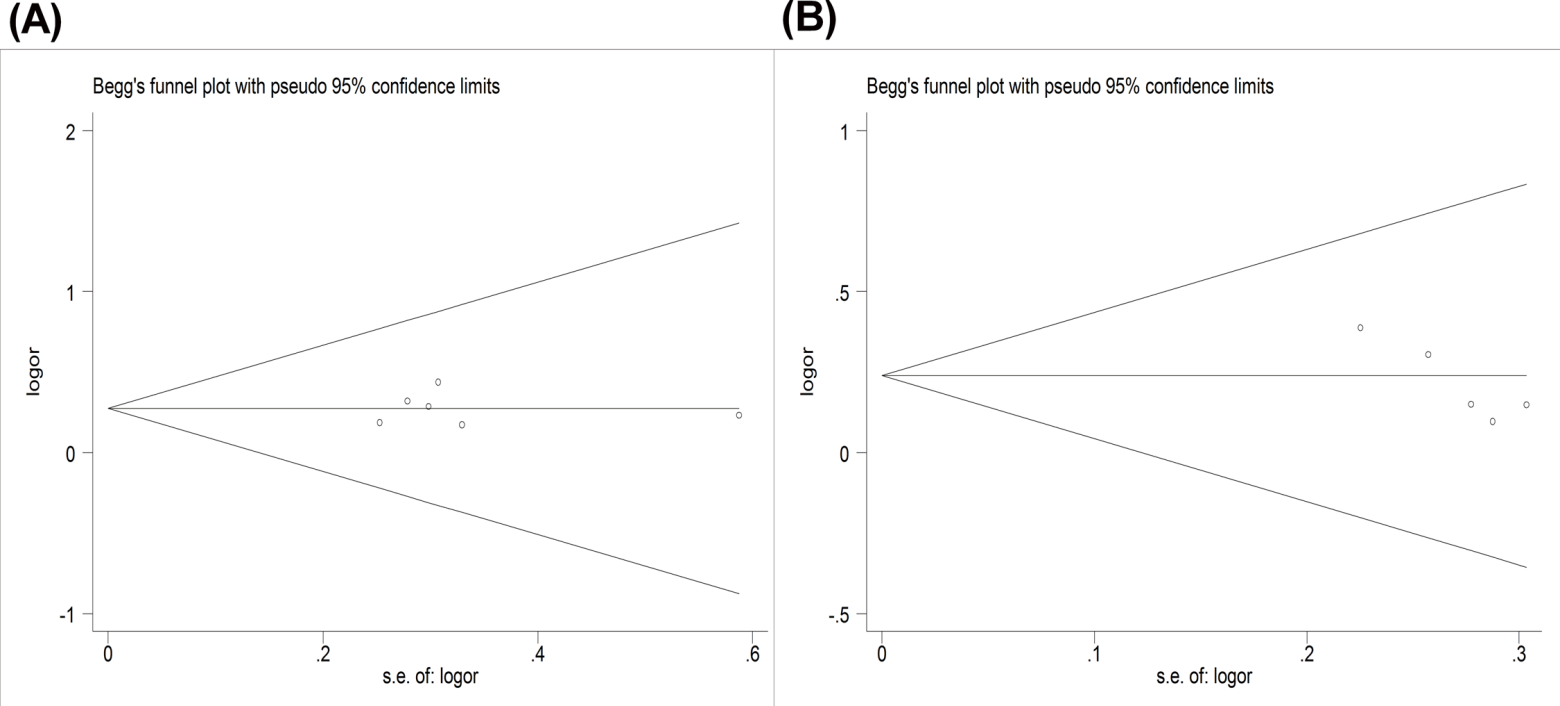
Begg’s funnel plot for analysis of publication bias for the IL-1α (+889C/T) and IL-1β (+3954C/T) polymorphisms for overall comparison (C versus T).

No heterogeneity was found for either polymorphism. Similarly, funnel plots and the Egger test showed no evidence of publication bias (Egger’s regression test *P* > 0.1) (Fig 5).

## Discussion

The major processes involved in intervertebral disc degeneration remain unclear; however, various intrinsic and extrinsic factors such as mechanical overloading, senescence, and environmental factors all contribute to pathological changes in the disc [33]. IDD is now recognized as a multifactorial disease, with an important genetic component [34, 35]. Many polymorphisms in genes including collagen, interleukins, and aggrecan, have been shown to be related to the risk of IDD [36]. Among the implicated genes, IL-1 is one of the most relevant and widely studied. In our seven eligible studies [14, 16–21], we demonstrated an increased risk of IDD among T allele variant carriers; however, conflicting results ranging from strong links to no association were obtained. As far as we know, our study is the first meta-analysis to address association between IDD and the IL-1α (+889C/T) or IL-1β (+3954C/T) polymorphisms. In this meta-analysis, a total of five and six studies, respectively, were ultimately included in the analyses for IL-1α (+889C/T) and IL-1β (+3954C/T). The IL-1α (+889C/T) polymorphism was associated with a statistically increased risk of IDD in heterozygote, dominant, and allelic models. As goodness-of is fit for the fixed and random effect models with assumption of normal distributions in meta-analysis. Goodness-of-fit test was applied to check the model adequacy [29], and there was strong evidence that (all studies/data) fit the model very well.

Moreover there was a significant association in a Caucasian subgroup after stratification by ethnicity (TT versus CC: OR = 2.95, 95% CI: 1.45, 6.04; *P*_heterogeneity_ = 0.82; TT versus CC/CT: OR = 2.29, 95% CI: 1.18, 4.47; *P*_heterogeneity_ = 0.20), in which the 95% CI did not overlap the lines of the pooling results. In contrast, we found that the IL-1β (+3954C/T) polymorphism was not significantly associated with the risk of IDD. In subgroup analysis, we demonstrated significant results in Asians using only a heterozygote model (CT versus CC: OR = 0.61, 95% CI: 0.38, 0.98; *P*_heterogeneity_ = 0.40) and a dominant model (CT/TT versus CC: OR = 0.60, 95% CI: 0.38, 0.96; *P*_heterogeneity_ = 0.41). Given the important role of IL-1, it is reasonable that the IL-1α (+889C/T) and IL-1β (+3954C/T) polymorphisms may contribute to IDD susceptibility.

IL-1 is a potent inflammatory cytokine that plays a key role in IDD. As a master proinflammatory mediator, IL-1 is highly expressed in degenerative disc tissues, and promoted in inflammatory responses and matrix degradation-processes that accelerate the grade of disc degeneration [37]. Thus, the expression of IL-1 gene polymorphisms could be related to predisposition to IDD. In previous studies, the TT genotype of IL-1α (+889C/T),when compared with the CC genotype, was significantly associated with higher transcriptional activity of this gene in plasma [38]. Besides, this polymorphism may affect the production of IL-1β: a higher level of IL-1β was detected in individuals with the TT genotype than in individuals with the CC genotype [39]. The common IL-1β (+3954C/T) polymorphism is associated with increased IL-1 levels [40]. In addition, the levels of other markers of systemic inflammation, such as C-reactive protein, are affected by the IL-1β (+3954C/T) polymorphism [41]. The T allele of the IL-1α (+889C/T) polymorphism was significantly more common among people with IDD than among control subjects. Serrano et al. reported no association between this polymorphism and IDD in the Mexican population; these results clearly conflict with those of other studies. After this study was removed, a significant association was reached under all genetic models. For the IL-1β (+3954C/T) polymorphism, although a trend towards increased risk of IDD could be seen in several Caucasian studies, none reached significance. In contrast, the two studies in Asians showed a different result: the IL-1β (+3954C/T) polymorphism had no effect on IDD risk. A different cohort and a different ethnic group may contribute to different results. Therefore, a real association between the IL-1β (+3954C/T) polymorphism and the risk of IDD cannot be excluded.

This is the first meta-analysis addressing the association between IL-1 gene polymorphisms and risk of IDD, but our study does have some limitations that require consideration. First of all, the small number of original studies, and the unpublished data that the authors did not provide, limit further analysis. Secondly, although the Begg’s funnel plot and Egger’s test refuted publication bias, the possibility of bias cannot be fully eliminated because only English and Chinese published studies were retrieved. Thirdly, because IDD is a multifactorial disease, the clinical effects of IL-1 gene polymorphisms might be magnified or masked by gene-gene and/or gene-environment interactions. Unfortunately we could not address this here because individual data were not available for other covariates such as age, family history, and environmental factors. Fourth, the results of the association between the IL-1β (+3954C/T) polymorphism and the risk of IDD remained inconclusive, even showing opposing trends in Asians and Caucasians. Ethnicity and the small amount of data available could explain this.

In conclusion, our results suggest that the IL-1α (+889C/T) polymorphism is significantly associated with increased susceptibility to IDD, particularly in Caucasians. In contrast, the IL-1β (+3954C/T) polymorphism may not be associated with the risk of IDD in Asians, while there is a clear trend towards association in Caucasians. Because of the limitations of this study, larger-scale studies in more populations, with consideration of gene-gene and/or gene-environmental interactions, are necessary to further explore the roles of IL-1 in the pathogenesis of IDD.

## Supporting Information

S1 PRISMA PRISMA IL-1. (PDF)
